# LC-MS/MS Detection of Tryptophan, Kynurenine, Kynurenic Acid, and Quinolinic Acid in Urine Samples from Drug-Positive and Illicit Drug-Negative Patients with a Known History of Substance Use Disorder

**DOI:** 10.3390/metabo15110749

**Published:** 2025-11-18

**Authors:** Lindsey Contella, Christopher L. Farrell, Luigi Boccuto, Alain H. Litwin, Hunter Flanagan, Stacy E. F. Melanson, Nicole V. Tolan, Marion L. Snyder, Dina N. Greene

**Affiliations:** 1 Healthcare Genetics and Genomics, School of Nursing, Clemson University, Clemson, SC 29631, USAlboccut@clemson.edu (L.B.); 2 Luxor Scientific, LLC, Greenville, SC 29607, USA; 3 School of Health Research, Clemson University, Clemson, SC 29631, USA; 4 Department of Medicine, Prisma Health, Greenville, SC 29605, USA; 5 Department of Medicine, University of South Carolina School of Medicine, 876 W Faris Rd., Greenville, SC 29605, USA; 6 Department of Pathology, Brigham and Women’s Hospital, Harvard Medical School, Boston, MA 02115, USA; semelanson@bwh.harvard.edu (S.E.F.M.);; 7 Department of Laboratory Medicine and Pathology, University of Washington, Seattle, WA 98195, USA

**Keywords:** tryptophan, kynurenine, serotonin, LC-MS/MS, urine, substance use disorder

## Abstract

Introduction: Currently, there are few tools for monitoring recovery in substance use disorder. As substance use has increased in prevalence, tools for measuring recovery are needed to improve therapeutic outcomes. Measuring the kynurenine pathway for imbalances in metabolites could be a possible solution to monitor recovery. Methods: We developed a liquid chromatography–tandem mass spectrometry (LC-MS/MS) method to quantify tryptophan, kynurenine, kynurenic acid, and quinolinic acid in urine. Metabolites were separated using a stepwise gradient and detected with an Agilent 6460 triple quadrupole mass analyzer. The samples were extracted using a simple protein precipitation protocol. Method validation was performed using routine toxicology urine samples and laboratory contrived samples. The performance characteristics assessed included precision, linearity, stability, interference, and matrix effects. Additionally, urine samples from two cohorts (illicit drug-negative and drug-positive; *n* = 120 per cohort) were analyzed for significant concentration differences in the four metabolites using Mann–Whitney, PCA, and Area Under the Receiver Operating Characteristic Curve statistical analysis. Results: The LC-MS/MS assay was linear from 195 to 100,000 ng/mL for tryptophan, 6 to 3000 ng/mL for kynurenine, 14 to 7200 ng/mL for kynurenic acid, and 125 to 64,000 ng/mL for quinolinic acid using an 8-point calibration curve. Imprecision ranged from 1.17% to 12.46% CV using two controls that spanned the analytical measurement range. Matrix effects were observed; however, the use of labeled internal standards matching the metabolites of interest minimized the impact on quantification. The extraction recovery efficiency was acceptable for the analytical validation. Ambient stability extended to 10 days, resulting in individual sample biases of up to 22%. A statistically significant increase in TRP, KYN, and QA was observed in drug-positive urine compared to illicit drug-negative urine (*p* < 0.01). Conclusion: We developed a rapid and sensitive LC-MS/MS method for quantifying tryptophan, kynurenine, kynurenic acid, and quinolinic acid in urine that can aid in future research elucidating the relationship between substance use disorders and tryptophan metabolism.

## 1. Introduction

Substance use disorder (SUD) is a complex and evolving public health crisis, characterized by rising rates of overdose deaths and misuse of prescription medications. Effective diagnostic, monitoring, and treatment tools to aid in substance use recovery are lacking [1]. Over the last 30 years, there has been an annual increase in SUD, also referred to as drug use disorder (DUD), that is projected to continue through 2035 [2]. Identifying biomarkers suitable for evaluating treatment efficacy could alleviate the ability to predict relapse and/or objectively assess treatment effectiveness.

Tryptophan (TRP) metabolism was recently identified as a mechanistic target of SUD [3]. The majority (95%) of TRP is metabolized through the kynurenine (KYN) pathway, which is known to influence numerous physiological systems, including inflammation, immune tolerance, neurodegeneration, and metabolic disease [4]. SUD is one of the newest disorders associated with the KYN pathway. A minor portion (5%) of TRP is metabolized through the serotonin pathway, which has previously been found to be disrupted in SUD; pharmacological manipulation of the serotonergic pathway could also aid in recovery from SUD [5]. The serotonin pathway has been extensively studied in relation to SUD but is not discussed here [6]. The serotonin pathway is tissue specific and only occurs in the enterochromaffin cells of the gut and serotonergic neurons of the raphe nuclei in the brain, highlighting the need for further research on the relationship between SUD and the KYN pathway [7].

**Figure 1:**
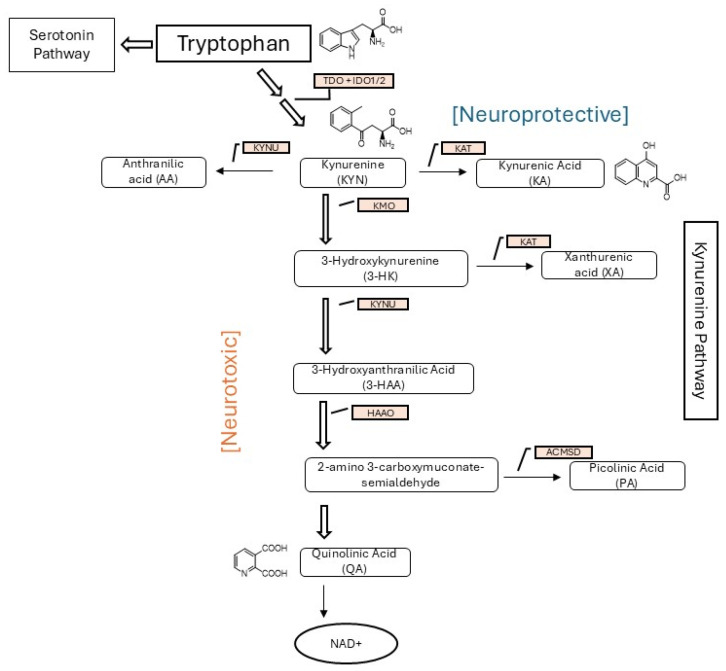
Metabolism of TRP by the kynurenine pathway showing the two main branches: the neurotoxic pathway and the neuroprotective pathway. Abbreviations: TDO: indoleamine 2,3-dioxygenase-1; IDO1/2: indoleamine 2,3-dioxygenase-1/2; TDO: tryptophan 2,3-dioxygenase; KATs: kynurenine aminotransferases; KMO: kynurenine 3-monooxygenase; KYNU: kynureninase; NE: nonenzymatic; HAAO: 3-hydroxy anthranilate 3,4-dioxygenase; NAD+: nicotinamide adenine dinucleotide.

TRP metabolism (Figure 1) is initiated by the enzyme tryptophan 2,3-dioxygenase (TDO) in the liver or indoleamine 2,3-dioxygenase (IDO1/2) in the immune system and brain, converting TRP to KYN [3]. This is the rate-limiting step of the KYN pathway, which is influenced by factors like cytokines and inflammation. The pathway then splits into neuroprotective and neurotoxic branches. The neuroprotective branch converts KYN to kynurenic acid (KA) via kynurenine aminotransferase (KAT). The neurotoxic branch involves KYN conversion to 3-hydroxykynurenine (3-HK), ultimately producing quinolinic acid (QA), which is further converted to NAD+, a key coenzyme.

Physiological maintenance of relative KA and QA concentrations is important; dysregulation of TRP metabolism has been implicated in multiple disease states, including neurodegenerative disorders, psychiatric conditions, immune-related disorders and, recently, SUD [8,9,10]. Monitoring the concentrations of the intermediates in the neuroprotective KA and neurotoxic pathway, from 3-HK to QA, could be used as a tool in SUD treatment.

We describe a method for sensitive and accurate determination of four (4) metabolites of the KYN pathway in urine: TRP, KYN, KA, and QA (Figure 1). Recent analytical advances have focused on developing profiling techniques for tryptophan metabolites in biological matrices, particularly urine, using mass spectrometry–based platforms. Among these, LC–MS/MS has become the preferred approach due to its high sensitivity, selectivity, and simpler sample preparation compared to GC–MS, allowing for the simultaneous quantification of multiple kynurenine pathway metabolites in complex biological samples. The goal of this study was to (1) develop a targeted, metabolomic liquid chromatography–mass spectrometry/mass spectrometry (LC-MS/MS) method to quantitatively detect TRP, KYN, KA, and QA in urine; (2) validate the method using the Clinical Lab Standards Institute (CLSI) standard for liquid chromatography–mass spectrometry methods (CLSI-C62) and the US FDA’s bioanalytical method validation guidance for industry; and (3) identify if a metabolic pattern exists for these metabolites in drug-positive compared to drug-negative SUD samples [11,12].

## 2. Materials and Methods

### 2.1. Preparation of Standards

Stock solutions of each deuterated internal and analyte standard were prepared based on their solubilities to a concentration of 1 mg/mL: L-TRP and QA in methanol, and L-KYN and KA in 1 M HCl. Analytical-grade methanol (MeOH) and 1 M hydrochloric acid solution (HCl) were obtained from Sigma-Aldrich (St. Louis, MO, USA). The standards were stored at −20 °C and were diluted in methanol before use to achieve the desired concentrations for calibrator, quality control, and working internal standard solutions.

L-KYN, L-TRP, KA, 2,3-pyridine-dicarboxylic acid (QA), and TRP indole-d5 were purchased from Sigma-Aldrich (St. Louis, MO, USA). KYN-d4 and KA-d5 were purchased from Cayman Chemical Company (Ann Arbor, MI, USA). QA-d3 was purchased from MedChemExpress (Princeton, NJ, USA).

### 2.2. Extraction Procedure

A Tecan XEVO (Tecan Group Ltd., Mannedorf, Switzerland) was used to pipette 50 µL of each urine sample plus 20 µL of IS solution into a 96-well plate. The plate was vortexed for 30 s and centrifuged at 10,000× *g* for 10 min. Next, protein precipitation was performed by transferring 50 µL of supernatant into a new 96-well plate followed by 50 µL of MeOH; the mixture was vortexed for 30 s and centrifuged for 5 min at 10,000× *g*. The supernatant (80 µL) was transferred to a second 96-well plate and was dried under nitrogen at 22 °C for 20 min before reconstituting in 50 µL of 1 mM ammonium acetate.

### 2.3. Analyte Separation and Instrument Acquisition Parameters

Separation and detection relied on an Infinity 1260 HPLC system coupled to a 6460 triple quadrupole mass spectrometer (Agilent Technologies, Santa Clara, CA, USA). Chromatographic separation was accomplished using a Waters Acquity UPLC BEH C18 (100 × 2.1 mm, 1.7 µm) reversed-phase column with mobile phase A (1 mM ammonium acetate in water, *v*/*v*) and B (MeOH). Ammonium acetate was obtained from Fisher Chemical (Pittsburgh, PA, USA). De-ionized water was supplied using a Millipore Milli-Q Direct 8UV purification system from Millipore Sigma (Burlington, MA, USA). The column temperature was set to 40 °C. Gradient elution was performed as follows: 5% to 60% B for the 0.0–5.0 min interval, 60% to 90% B from 5.0 to 5.01 min, 90% B from 5.01 to 6.0 min, 90% to 5% B from 6.0 to 6.01 min, and held at 5% from 6.01 to 7.0 min (Supplementary Table S1). The autosampler temperature was set to 12 °C. The flow rate and injection volume were set to 0.25 mL/min and 10 µL, respectively.

Samples were analyzed using electrospray ionization (ESI) in positive-ion multiple-reaction-monitoring (MRM) mode. The mass spectrometric conditions were set as follows: gas flow of 10 L/min; nebulizer pressure of 35 psi; sheath gas heater temperature of 300 °C; sheath gas flow of 10 L/min; capillary voltage of 4000 V; nozzle voltage of 500 V; and Delta EMV of (+) 300 V. Quantitative and qualitative MRM transition ions were selected because they were the most abundant product ions. The optimized MRM parameters are summarized in Table 1. Data acquisition and analysis of all MRM chromatograms were performed using MassHunter B.08.00 software (Agilent Technologies).

The optimization of the mass spectrometric parameters was evaluated by injecting a 10,000 ng/mL working solution at a flow rate of 0.250 µL/min. The quantitative ion pairs selected for TRP, KYN, KA, QA, TRP-D5, KYN-D4, KA-D5, and QA-D3 were 205.2 ˃ 188, 209.2 ˃ 192, 190.2 ˃ 144, 168.1 ˃ 124, 210.3 ˃ 192.1, 213.2 ˃ 96.1, 195.2 ˃ 149, and 171.1 ˃ 153, respectively. The qualifier ion pairs selected for TRP, KYN, KA, and QA were 205.2 ˃ 118, 209.2 ˃ 94.1, 190.2 ˃ 89, and 168.1 ˃ 150, respectively. The chromatographic retention time of TRP, KYN, KA, and QA were 3.318, 2.447, 3.535, and 1.007, respectively, for a total run time of 8 min (Table 1).

### 2.4. Samples

Urine samples received for routine drug testing at Luxor Scientific, LLC (Greenville, SC, USA), a reference laboratory, were used for method validation. The participants were provided instructions from a trained laboratory assistant before collecting an unobserved, randomly timed urine sample. The samples were evaluated for 96 illicit and prescription drugs using a validated clinical LC-MS/MS assay performed at Luxor Scientific, LLC. Illicit drug-negative (*n* = 120) and drug-positive (*n* = 120) cohorts were defined based on the following criteria.

Samples were included in the illicit drug-negative cohort if they were negative for all substances except cotinine. Samples positive for cotinine were statistically indistinguishable using Mann–Whitney statistical test from those that were negative, supporting their inclusion. No ICD-10 limitations were applied to this cohort.

Samples were included in the drug-positive cohort if they were positive for one or more of the following highly addictive substances: cocaine (*n* = 78), methamphetamine (*n* = 39), fentanyl (*n* = 15), and/or xylazine (*n* = 8). Additionally, all drug-positive samples had an ICD-10 code indicating SUD (F11-F19).

Urine creatinine concentration (mg/dL) was measured using the Jaffe reagent (Thermo Fisher, Waltham, MA, USA) and the concentration in each sample was used to normalize the concentration of the four TRP metabolites to the overall solute concentrate of the urine sample. Samples were de-identified, stored at RT, and processed within 72 h to measure TRP, KYN, KA, and QA. Institutional Review Board (IRB) was obtained at Clemson University (IRB 2024-0421).

### 2.5. Method Validation

The TRP, KYN, KA, and QA urine quantification methods were validated following the bioanalytical guidelines provided by the US FDA and the Clinical and Laboratory Institute (CLSI) document C62 Liquid Chromatography–Mass Spectrometry Methods [11,12].

#### 2.5.1. Percent of Expected Concentration and Precision

The percent of expected concentration was evaluated by measuring two levels of QC, low and high levels, with concentrations (ng/mL) of 1500 and 10,000 for TRP, 200 and 2500 for KYN, 500 and 7000 for KA, and 625 and 5000 for QA. QC was run in triplicate over five days and compared to the expected concentration.

Intra-day precision was determined by running two levels of QC (8 wells per level) and injecting each well in triplicate. Inter-day precision was determined by running two levels of QC in a single well and injecting each well in triplicate over ten days. Acceptability criteria for precision were within ±15% CV, and the percentage of expected concentration acceptability was ±15% of the expected concentration.

Incurred sample reanalysis (ISR) was performed by testing ten patient samples on two separate days to assess precision further; these were acceptable if the two runs were within a ±20% bias.

#### 2.5.2. Linearity

Linearity was confirmed using an 8-point calibration curve. Calibrator concentrations (ng/mL) for TRP were 195, 391, 781, 1563, 6250, 25,000, 50,000, and 100,000; for KYN, they were 6, 12, 24, 48, 375, 750, 1500, and 3000; for KA, they were 14, 28, 56, 113, 900, 1800, 3600, and 7200; and for QA, they were 125, 250, 500, 1000, 8000, 16,000, 32,000, and 64,000. Each calibrator was run in triplicate over four days. The standard curve was calculated using the ratio of the peak area of each metabolite to its corresponding IS. Acceptance limits were ±15% of the expected concentration, except for the lowest-concentration calibrator (LLOQ) which had a ±20% bias (acceptable). Data evaluation was completed using the EP Evaluator Linearity Module (12.3.02).

#### 2.5.3. Analytical Sensitivity

The limit of detection (LOD) was determined by serially diluting the lowest-concentration calibrator and defined as the lowest concentration yielding a signal-to-noise (S/N) of five or greater. The LOD was verified by injecting a sample containing all four analytes (6 ng/mL TRP, 1 ng/mL KYN, 1 ng/mL KA, and 4 ng/mL QA) in triplicate on three separate runs. The limit of quantification (LOQ) was calculated at 10 times the standard deviation (SD) of the injected LOD samples.

#### 2.5.4. Carry-Over

The highest-concentration calibrator containing all four analytes (100,000 ng/mL TRP, 3000 ng/mL KYN, 7200 ng/mL KA, and 64,000 ng/mL QA) was used to evaluate the carryover by injecting a negative sample, followed by the highest-concentration calibrator and then three negative samples; the acceptability criterion was that the negative sample should not exceed 20% of the LLOQ.

#### 2.5.5. Matrix Effects

Matrix effects (MEs) were evaluated as previously described by Matuszewski et al. [13]. Ion suppression/enhancement for TRP, KYN, KA, and QA were assessed by creating three samples containing 50 µL of “sample” with 20 µL of working internal standard. The first sample was spiked with a contrived sample at 75% of the upper limit of linearity for each analyte (Sample 1). The second sample was a urine sample spiked with the contrived sample stated in Sample 1 (Sample 2). The third sample was the same urine sample used for Sample 2, but run neat (Sample 3). The ratio of concentrations of the analyte to internal standard was used to determine the overall matrix effects (comparison of Sample 2 to Sample 1).

Additionally, the CV of the internal standard signal for each calibration level, QC level, and patient sample (*n* = 10) for a typical run was plotted to determine if ion suppression was observed.

Extraction recovery was determined by calculating the concentration difference between QC samples that had completed the extraction process to QC samples added directly to a post-extraction blank using the following equation: (response of metabolites in pre-extraction sample/response of metabolites in post-extraction QC sample) × 100 (%).

#### 2.5.6. Interference Assessment

Proteinuria interference was assessed by pipetting 10 µL of 1% BSA into 40 µL of a urine sample and pipetting 10 µL of 1% BSA into a sample made up of water to determine potential false-positive or false-negative results. Each sample was injected once.

To assess the interference from blood, 5 µL of whole blood was mixed with 45 µL of urine to determine if blood would alter the urine results compared to the unaltered urine sample. Blood (10 µL) was mixed with water to see if it could cause a false-positive result.

A toxicology quality control sample was run one time as a negative control to assess if 96 medications or illicit substances would cause false-positive results (Supplementary Table S2). The quantifier and qualifier retention times for TRP, KYN, KA, and QA were evaluated for peaks.

#### 2.5.7. Stability

The stability of TRP, KYN, KA, and QA in the urine samples was monitored over a 10-day period. An aliquot was stored in the refrigerator (2–8 °C) and a second aliquot was stored at room temperature (22–25 °C). Ten patient samples were run on day 1 and day 10.

#### 2.5.8. Data Analysis

Agilent MassHunter B.08.00 was used to evaluate the chromatographic data. Microsoft Office Excel 2010 was used to calculate bias, standard deviations, and coefficients of variation (CVs). EP Evaluator (Build 12.3.02) was used to determine if the linearity of the calibrators was acceptable. R Studio (VER2024.04.2 Build 764) was used to conduct a statistical analysis of the SUD patient samples compared to the control cohort samples to determine if the concentrations of the metabolites were statistically significantly different between the cohorts. Mann–Whitney tests were performed to assess the statistical significance. It was considered statistically significant if the *p*-value was less than 0.05. Receiver operation characteristics (ROCs) and their area under the curve (AUC) were used to determine the sensitivity and specificity of the two cohorts. GraphPad Prism (VER 10.3.0) was used to construct graphs and perform AUC and ROC statistical analysis. Additionally, principal component analysis (PCA) was performed on normalized KYN pathway metabolite concentrations (KYN, TRP, QA, and KA) to determine the variance between the illicit drug-negative and drug-positive cohorts. The analysis was conducted using Python (VER 3.12) in Google Colab.

## 3. Results

### 3.1. Percent of Expected Concentration and Precision

Across five runs (*n* = 3/level/run), the total imprecision ranged from 1.17% to 12.46% CV, and the percent of expected concentration ranged from −11.10% to 9.32% relative to the expected concentrations, as shown in Table 2. Reanalysis of the patient samples (ISR) was acceptable, with the results from the repeated patient samples being within 20% bias of their initial run.

### 3.2. Linearity

The assay was linear over the analytical measuring range (AMR) (Supplementary Figure S2). A 1/x weighting factor forced through y = 0 was applied to achieve optimal precision and accuracy. All analytes’ standard curves had a regression coefficient (r^2^) > 0.99 (Table 2).

### 3.3. Analytical Sensitivity

The LOD was determined to be 6 ng/mL for TRP, 1 ng/mL for KYN, 1 ng/mL for KA, and 4 ng/mL for QA. The LOD was supported by the illicit drug-negative and drug-positive cohorts as all 240 samples had concentrations of these metabolites above the LOD. The chromatograms of the LOD samples are shown in Supplementary Figure S2. The LOQ was determined to be 16.8, 1.61, 2.84, and 11.8 ng/mL for TRP, KYN, KA, and QA, respectively.

### 3.4. Carry-Over

Carry-over was negligible at <0.05% for all analytes (Supplementary Table S3).

### 3.5. Matrix Effects

The mean percentage matrix effects were 295% for TRP, 332% for KYN, 102% for KA, and 315% for QA. However, when analyzing the final concentration of the matrix samples using IS normalization, the CV ranged from 5 to 9%. The average concentration of the pre-spike sample was 17,543 compared to 15,170 ng/mL for TRP, 2341 compared to 2545 ng/mL for KYN, 731 compared to 621 ng/mL for KA, and 7641 compared to 6774 ng/mL for QA. Extraction recovery was acceptable with 106%, 107%, 97%, and 100% recovery percentages for TRP, KYN, KA, and QA, respectively. Table 2 summarizes the observed matrix effects and extraction recovery.

The CV of the internal standard across a representative run of calibration (*n* = 8), QC (*n* = 2) level, and patient sample (*n* = 10) was <30% for each analyte.

### 3.6. Interference Assessment

Excess albumin did not interfere with detection. The bias between the neat urine sample and the sample with BSA was 1% for TRP, −1% for KYN, −6% for KA, and −1% for QA. The BSA–water matrix had concentrations of all metabolites below the LLOQ (Supplementary Table S4).

Blood did not significantly interfere with the detection of the metabolites. The sample containing water mixed with blood did not produce a false-positive result. The bias between the neat urine sample and the sample with blood was −1%, 6%, 1%, and 9% for TRP, KYN, KA, and QA, respectively (Supplementary Table S4.

QC testing of the comprehensive toxicology (Supplementary Table S2) did not produce a visible peak for any of the four metabolites (TRP, KYN, KA, or QA).

### 3.7. Stability

The percentage differences [(initial concentration-repeat concentration)/initial concentration] for the samples stored at ambient temperature for up to 10 days resulted in individual sample biases of up to 22%, as shown in Table 3.

### 3.8. Clinical Performance

**Figure 2:**
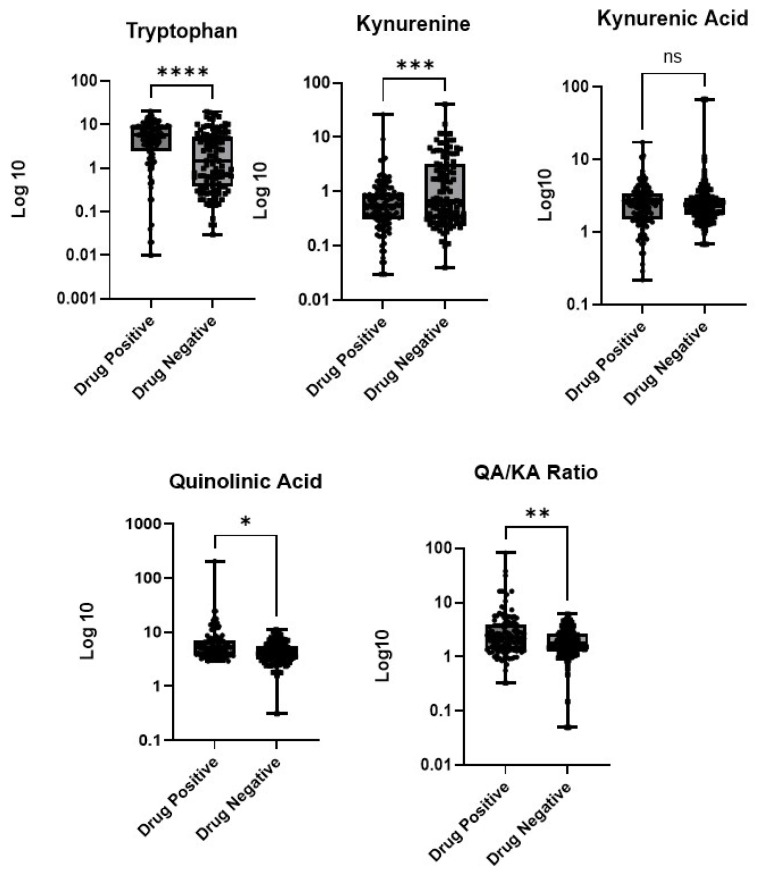
Boxplot of concentrations (µg/mg) of tryptophan, kynurenine, kynurenic acid, and quinolinic acid in the samples from the illicit drug-negative (*n* = 120) and drug-positive cohorts (*n* = 120). * *p* < 0.05, ** *p* < 0.005, *** *p* < 0.001, **** *p* < 0.0005, and ns = not significant.

**Figure 3:**
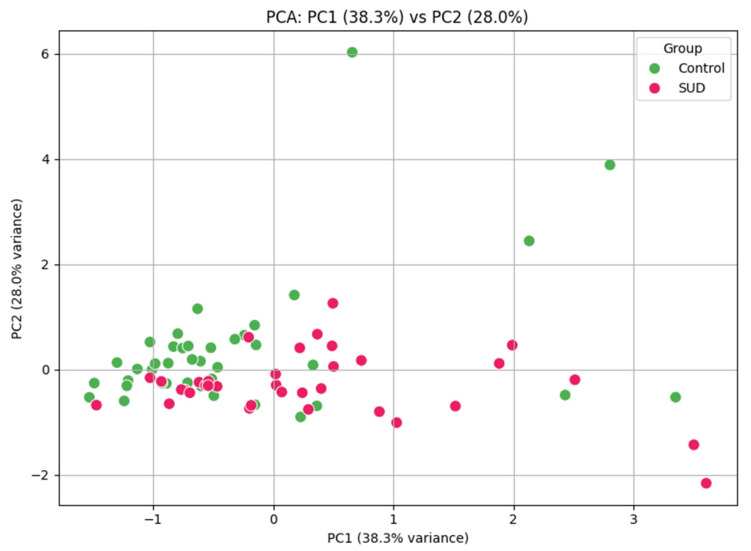
Principal component analysis (PCA) of the KYN pathway metabolites tryptophan, kynurenine, kynurenic acid, and quinolinic acid. The first two principal components explain 66.3% of the variance. Control samples cluster tightly, while the SUD samples are more dispersed.

Urine samples from the illicit drug-negative cohort (*n* = 120) were compared to those from the drug-positive cohort (*n* = 120) (Figure 2). The samples ranged in concentration from 228 to 56,887 ng/mL for TRP, 38 to 50,215 ng/mL for KYN, 79 to 81,450 ng/mL for KA, and 292 to 343,389 ng/mL for QA. Normalizing the results to the urine creatinine concentration resulted in a median concentration of 1.49 and 5.75 µg/mg for TRP in the illicit drug-negative cohort and positive cohorts, respectively (*p* < 0.0001); the median KYN concentrations were 0.547 and 0.674 µg/mg for the illicit drug-negative and positive cohorts, respectively (*p* < 0.0028); the median KA concentrations were 2.212 and 2.518 µg/mg for the illicit drug-negative and positive cohorts, respectively (*p* = 0.489); and the QA concentrations were 4.086 and 5.116 µg/mg for the illicit drug-negative and positive cohorts, respectively (*p* < 0.0001) (Table 4). The area under the ROC curve was calculated for each analyte, and the results of the illicit drug-negative cohort and drug-positive cohort were compared. The results showed moderate discrimination for QA and KYN, with AUROCs of 0.716 (*p* < 0.001) and 0.746 (*p* < 0.001), respectively. The ROC curve analysis demonstrated minimal discriminatory ability, with AUROC values of 0.562 and 0.534, respectively. PCA of the four metabolites of the KYN pathway (QA, KA, TRP, and KYN) revealed separation between the two cohorts. The first two principal components accounted for 66.3% of the variance. The control samples clustered tightly, while the SUD samples showed greater dispersion, suggesting that SUD is associated with altered KYN metabolism (Figure 3).

Using the reference intervals published by Oh et al., 6%, 11%, and 25% of the illicit drug-negative cohort and 6%, 35%, and 26% of the drug-positive cohort had elevated TRP, KYN, and KA levels, respectively (Table 5). Using the reference intervals published by Gunn et al., 21% of samples in the illicit drug-negative cohort and 36% of the samples in the drug-positive cohort had an elevated QA concentration [10,14]. We also observed statistically significant differences between the ratio of QA:KA between the drug-positive and drug-negative cohorts (Figure 2).

## 4. Discussion

Here, a rapid (8 min) LC-MS/MS method was developed to detect four metabolites associated with TRP metabolism in urine. The method was validated following standard protocols to ensure adequate analytical sensitivity and specificity. All metabolites exhibited linearity appropriate for the clinically observed concentration range, and the intra- and inter-day precision confirmed consistency. The method uses a simple preparation process while maintaining the clinically necessary limit of detection (LOD) and limit of quantification (LOQ).

The metabolites analyzed using this method were chosen because of their varied roles in disease. QA is a neurotoxin that has been implicated in the pathogenesis of neurodegenerative disorders, such as Parkinson’s disease [15]. The ability to accurately measure QA could facilitate the early diagnosis and the monitoring of disease progression. KYN and KA have been associated with immune regulation and neuroprotection. Imbalances in their systemic concentration have been linked to psychiatric conditions such as depression and schizophrenia [16]. A relatively simple and fast method like the one described herein to measure these metabolites could advance our understanding of their roles in these disorders and guide therapeutic interventions. While this study focused on four key metabolites within the kynurenine pathway, we acknowledge that inclusion of additional intermediates such as 3-hydroxykynurenine, anthranilic acid, and picolinic acid would provide a more comprehensive understanding of pathway alterations. Future work expanding metabolite coverage will be essential to strengthen the biological interpretation and fully characterize kynurenine pathway dynamics in substance use disorders.

The successful detection and quantification of TRP, KYN, QA, and KA have implications for understanding the role of the KYN pathway in health and disease. This method was applied to a subset of clinical urine samples to determine if patients with SUD have altered KYN pathway metabolism compared to a population that was negative for both illicit and prescription drugs. Limited research has evaluated KYN pathway metabolites in urine as it relates to SUD. Only three studies have analyzed KYN pathway metabolites in human urine samples, which is a preferable sample type due to its inherent concentration of small molecules/metabolites and the routine collection of this sample type from patients with SUD. These studies did not evaluate QA or KA concentrations, and the studies measuring TRP and KYN were limited to alcohol and opioid use disorder patients [17,18,19]. Maciejczyk et al. evaluated the TRP and KYN levels in post-mortem urine samples of patients with acute alcohol intoxication compared to a healthy cohort. The study identified a significant difference in TRP concentrations. This study did not observe the same finding, which could be due to the difference in the SUD groups; this study did not evaluate alcohol as an illicit substance or the potential changes that occur post-mortem. Previous work has shown that systemic concentrations of QA and KA are altered in blood and brain samples from both human and animal SUD models [3].

Two review articles summarized multiple studies that evaluated animal and human samples that found QA, the neurotoxic metabolite, was upregulated compared to KA, the neuroprotective metabolite, causing an imbalance in the KA:QA ratio [3,9]. Morales-Puerto et al. discussed the association between the KYN pathway and alcohol, nicotine, cannabis, amphetamine, and cocaine use, indicating that there is a decrease in KA in cocaine and amphetamine use disorder patients and an increase in KA in nicotine use disorder patients. Additionally, the KYN concentration was increased in alcohol but decreased in amphetamine use disorders. They summarize the alterations in the KYN pathway due to substance use and identified how modulation of this pathway is a potential approach for therapeutic intervention.

The preliminary clinical evaluation identified a significant difference in metabolite concentration for QA and KYN between the two cohorts. The ROC analyses provided an initial assessment of KYN pathway metabolites in an illicit drug-negative cohort and drug-positive cohort. QA and KYN concentrations demonstrated moderate discriminatory abilities (AUROC of 0.716 and 0.746, both *p* < 0.001) when comparing the illicit drug-negative and drug-positive cohorts, suggesting that these metabolites need further investigation as potential biomarkers for monitoring treatment response in SUD. In contrast, TRP and KA concentrations showed minimal discrimination abilities (AUROC of 0.562 and 0.534), indicating that not all pathway metabolites contribute equally to classification performance. PCA of the KYN pathway metabolites identified differences in the metabolic profiles of the two cohorts. The illicit drug-negative cohort results were clustered, while the SUD cohort had greater variability. This result highlighted the dysregulation of the KYN pathway in SUD. The observable difference between the two cohorts supports the need to further investigate the utility of KYN pathway metabolites as objective biomarkers in SUD research.

Although these findings are preliminary, they support the feasibility of applying quantitative KYN pathway profiling in future studies aimed at developing objective biochemical markers to complement current clinical assessments. However, these results should be interpreted with the limited sample size and exploratory nature of the study in mind. A larger sample size and more in-depth research are needed to confirm this finding and to determine if a correlation truly exists for the metabolites TRP, KYN, KA, and QA with SUD. Additionally, more investigation is needed on the patterns of TRP metabolites in a comprehensive list of substance use disorders, including alcohol, opioids, methamphetamine, and cocaine disorders since the previous research is limited to almost exclusively alcohol disorders, with a minimal number of papers on disorders concerning other addictive substances. A method analyzing metabolite concentrations in oral fluid, which have been shown to mirror blood metabolite concentration, may provide further insight and help validate these preliminary findings. This additional research could further identify patterns in the metabolite profiles of patients with SUD compared to a control cohort.

An additional limitation of this study is that the role of diet and inflammation have on the pathway was not investigated. Tryptophan metabolism through the kynurenine pathway is highly sensitive to both dietary intake and inflammatory status. Only a small portion of dietary tryptophan is directed toward serotonin synthesis while the majority is metabolized via the kynurenine pathway. Under normal physiological conditions, dietary tryptophan availability influences the flux through this pathway; however, if inflammation occurs, it can play a more dominant regulatory role. Pro-inflammatory cytokines such as interferon-γ and tumor necrosis factor-α upregulate the rate-limiting enzymes indoleamine 2,3-dioxygenase (IDO1 and IDO2), shifting tryptophan metabolism toward increased production of kynurenine and downstream metabolites like quinolinic acid, which is neurotoxic, and away from neuroprotective metabolites such as kynurenic acid. This inflammatory modulation may contribute to the pathophysiology of neurodegenerative and psychiatric disorders, as well as immune dysregulation. Moreover, dietary factors such as protein intake and micronutrient status can further influence the activity of key enzymes in this pathway, including tryptophan 2,3-dioxygenase (TDO) in the liver. Future studies incorporating inflammatory markers and dietary information will be important in order to better contextualize these findings

Additional limitations of this study must be acknowledged. Accuracy was not fully accessed in this validation and, if used in a clinical setting, would need to be further evaluated. A small sample size was used for clinical validation. Ideally, a larger cohort would be monitored prospectively and longitudinally. Additionally, an area under the curve (AUC) analysis should be performed to further understand the diagnostic value and to define the optimal cut-offs. Matrix effects, while not found to impact the expected concentrations, were present. Matrix effects also impacted the internal standard concentration since the calibrators and quality control samples were not matrix-matched. Such high ion enhancement or suppression can arise from co-eluting endogenous compounds present in urine and may affect analytical robustness when applied across different laboratories or sample matrices. The use of isotopically labeled internal standards helped to correct for these effects within this study; however, variability in sample composition between populations or laboratories may still influence quantitative reproducibility. Alternative techniques such as solid-phase extraction, liquid–liquid extraction, QuEChERS, or matrix-matched calibration could be used to decrease the matrix effects. Future inter-laboratory validation and assessment in other biological matrices (e.g., plasma and saliva) will be essential to confirm the method’s transferability and ensure consistent performance in broader clinical applications. Prior to using this method for clinical purposes, the extraction method could be adjusted to further extract the sample and reduce the matrix effects. Another limitation is that the illicit drug-negative cohort did not exclude nicotine-positive patients and could ultimately skew the results since previous research had found nicotine to impact KA results. Future studies with more detailed behavioral and substance use data will be important for clarifying the specific effects of nicotine and other substances on pathway dynamics. The clinical samples were not screened for alcohol prior to inclusion in the illicit drug-negative cohort; as alcohol can affect the KYN pathway, it should be accounted for in future studies. Additionally, while our method was limited to four primary metabolites, there are others in the KYN pathway. For example, PA, another metabolite of the KYN pathway, was not evaluated in this study because there is no evidence that there is a relationship between SUD and PA. However, expansion of currently available methods could provide a more comprehensive understanding of the pathway and its relation to SUD. Additionally, although convenient, urine is not an ideal matrix for quantitative measurements due to its dependence on hydration and kidney function [20].

## 5. Conclusions

We developed and validated a rapid, accurate, and sensitive LC-MS/MS method for the simultaneous quantification of TRP and its related metabolites in urine samples within a single analytical run. The method demonstrated strong analytical performance, including high robustness, precision, and reproducibility, supporting its reliability for clinical and research applications. The initial application of this assay to clinical samples provided exploratory insights into potential alterations in kynurenine pathway metabolites; however, these preliminary findings require confirmation in larger and more diverse cohorts. Future studies should aim to expand the range of detectable metabolites and further validate the method across broader clinical contexts to strengthen the biological interpretation and translational relevance.

## Figures and Tables

**Table 1:** Optimized parameters for mass spectrometer detection of analytes and internal standards.

	TRP	KYN	KA	QA	TRP-D5	KYN-D4	KA-D5	QA-D3
CAS #	73-22-3	2922-83-0	492-27-3	89-00-9	62595-11-3	2672568-86-2	350820-13-2	138946-42-6
Molecular Weight	204.23	208.21	189.17	167.12	209.26	212.2	194.2	170.14
Precursor ion [*m*/*z*]	205.2	209.2	190.2	168.1	210.3	213.2	195.2	171.1
Quantifier Ion [*m*/*z*]	188	192	144	124	192.1	96.1	149	153
Qualifier Ion [*m*/*z*]	118	94.1	89	150	-	-	-	-
Collision Energy [eV]	5	5	17	5	5	5	17	5
Retention Time (RT) [Min]	3.318	2.447	3.535	1.007	3.29	2.411	3.459	0.97

**Table 2:** Method validation results for linearity, precision, extraction recovery, and matrix effect of tryptophan, kynurenine, kynurenic acid, and quinolinic acid.

										Low QC			High QC	
			ng/mL				ME%	Extraction Recovery (%)	% of Expected Concentration	CV%		Accuracy (%)	CV%	
Analyte	R^2^	Weight	LOD	Linear Range	Low QC	High QC				Intra-Day	Inter-Day		Intra-Day	Inter-Day
Tryptophan	0.997	1/x	6	98 to 100,000	1500	10,000	295%	106%	9.60%	6.34%	4.55%	5.50%	2.12%	10.25%
Kynurenine	0.999	1/x	1	3 to 3000	200	2500	332%	107%	−11.10%	1.17%	6.88%	−2.80%	4.49%	8.44%
Kynurenic Acid	0.999	1/x	1	7 to 7200	500	7000	102%	97%	−0.90%	1.55%	6.48%	−1.10%	3.33%	8.45%
Quinolinic Acid	0.998	1/x	4	63 to 64,000	625	5000	315%	100%	1.50%	9.32%	12.46%	−2.70%	2.41%	4.84%

Abbreviations: LOD, limit of detection; QC, quality control; ME, matrix effect; CV, coefficient of variation.

**Table 3:** Stability data for each metabolite after 1 and 10 days of being stored in a refrigerator and at room temperature.

Kynurenine	ng/mL			RF	RT	Tryptophan	ng/mL			RF	RT
Sample	Initial Run	RT	RF	% Bias	% Bias	Sample	Initial Run	RT	RF	% Bias	% Bias
Sample 1	4459	3907	3976	−11%	−12%	Sample 1	12,991	14,697	13,246	2%	13%
Sample 2	943	923	925	−2%	−2%	Sample 2	1611	1652	1602	−1%	3%
Sample 3	3356	3416	3433	2%	2%	Sample 3	6504	6619	6596	1%	2%
Sample 4	482	485	465	−3%	1%	Sample 4	21,940	22,405	22,843	4%	2%
Sample 5	5921	5830	5803	−2%	−2%	Sample 5	65	69	62	−5%	6%
Sample 6	6874	6745	6717	−2%	−2%	Sample 6	4908	4621	4616	−6%	−6%
Sample 7	952	967	1004	5%	2%	Sample 7	38,604	41,220	40,419	5%	7%
Sample 8	147	148	145	−1%	1%	Sample 8	13,030	12,460	12,833	−2%	−4%
Sample 9	151	147	148	−2%	−2%	Sample 9	2654	2672	2669	1%	1%
Sample 10	806	802	779	−3%	−1%	Sample 10	20	20	20	4%	5%
**Kynurenic Acid**				**RF**	**RT**	**Quinolinic Acid**				**RF**	**RT**
Sample	Initial Run	RT	RF	% Bias	% Bias	Sample	Initial Run	RT	RF	% Bias	% Bias
Sample 1	8570	6737	6718	−22%	−21%	Sample 1	3709	2883	2978	−20%	−22%
Sample 2	4479	4458	4446	−1%	0%	Sample 2	1930	1617	1600	−17%	−16%
Sample 3	785	778	796	1%	−1%	Sample 3	2821	2234	2253	−20%	−21%
Sample 4	10,853	9337	8854	−18%	−14%	Sample 4	6107	6063	5925	−3%	−1%
Sample 5	1208	1179	1189	−2%	−2%	Sample 5	13,052	12,027	14,536	11%	−8%
Sample 6	6260	6289	6300	1%	0%	Sample 6	8023	7037	6415	−20%	−12%
Sample 7	3861	3836	3906	1%	−1%	Sample 7	3321	3409	3403	2%	3%
Sample 8	2403	2391	2346	−2%	−1%	Sample 8	1219	1223	1199	−2%	0%
Sample 9	667	662	663	0%	−1%	Sample 9	8656	8569	8299	−4%	−1%
Sample 10	427	417	421	−1%	−2%	Sample 10	6460	5084	5179	−20%	−21%

Abbreviations: RT, room temperature; RF, refrigerated.

**Table 4:** The creatinine-adjusted concentrations (µg/mg) for the four metabolites in the drug-negative and drug-positive cohorts.

	Illicit Drug-Negative				Drug-Positive			
	TRP	KYN	KA	QA	TRP	KYN	KA	QA
Mean	3.64	2.66	3.61	4.58	6.27	1.02	2.89	13.41
Median	1.49	0.547	2.212	4.086	5.75	0.674	2.518	5.116
Minimum	0.03	0.04	0.68	0.31	0.01	0.03	0.22	1.93
Maximum	19.87	40.17	67.88	11.49	23.7	26.16	17.34	429.24
Standard Deviation	4.39	4.72	8.53	2.18	4.75	2.54	2.31	46.6

**Table 5:** Comparison of drug-negative and positive cohorts to published reference intervals.

Compound	Reference Interval (µg/mg)	% Illicit Drug Neg	% Drug Pos	Reference
TRP	8.38 +/− 5.35	6%	6%	[14] *
KYN	0.63 +/− 1.09	11%	35%	[14] *
KA	10.04 +/− 8.44	25%	26%	[14] *
QA	0.0–6.3	21%	36%	[10] ^#^

* Study population contained 163 controls. Samples were obtained in the morning after a 12 h fast. ^#^ Range established using a healthy population of donors who had no history of chronic pain or opioid use.

## Data Availability

Data available upon request.

## Supplementary Materials

The following supporting information can be downloaded at: https://www.mdpi.com/article/10.3390/metabo15110749/s1, Figure S1: Chromatograms of the peak at the limit of detection for each compound (TRP, KYN, KA and QA) in the method.; Figure S2: Linearity plots from EP Evaluator for each compound (TRP, KYN, KA and QA) in the method; Table S1: Instrument Gradient; Table S2: List of prescription and illicit compounds evaluated for interference.; Table S3: Results of the carry-over study.; Table S4: Results of the interference study to determine if blood or protein interfere with results of the assay.

## Abbreviations

The following abbreviations are used in this manuscript: TRP, tryptophan; SP, serotonin pathway; SUD, substance use disorder; KYN, kynurenine; 3-HAA, 3-hydroxyanthranilic acid, 3-HK, 3-hydroxykynurenine; QA, quinolinic acid; KA, kynurenic acid; AA, anthranilic acid; XA, xanthurenic acid; PA, picolinic acid; THP, tryptophan hydroxylase; AAAD, aromatic acid decarboxylase; IDO, indoleamine 2,3-dioxygenase; TDO, tryptophan 2,3-dioxygenase; KATs, kynurenine aminotransferases; KMO, kynurenine 3-monooxygenase; KYNU, kynureninase; NE, nonenzymatic; HAAO, 3-hydroxyanthranilate 3,4-dioxygenase; AFMID, arylformamidase; TCE, LC-MS/MS, liquid chromatography–tandem mass spectrometry; MeOH, methanol; HCL, hydrochloric acid; ESI, electrospray ionization; MRM, multiple-reaction monitoring; ISR, incurred sample reanalysis; LLOQ, lower limit of quantification; S/N, signal-to-noise; ME, matrix effect; CV, coefficient of variation.
