# Supplementary material for: LC-MS/MS Detection of Tryptophan, Kynurenine, Kynurenic Acid, and Quinolinic Acid in Urine Samples from Drug-Positive and Illicit Drug-Negative Patients with a Known History of Substance Use Disorder
Source: Metabolites. 2025 Nov 18;15(11):749. doi: 10.3390/metabo15110749 (PMC12654100; doi:10.3390/metabo15110749)
Supplement: Supplementary file 1 [file metabolites-15-00749-s001.zip › metabolites-3915710-supplementary.pdf]

Supplementary Data

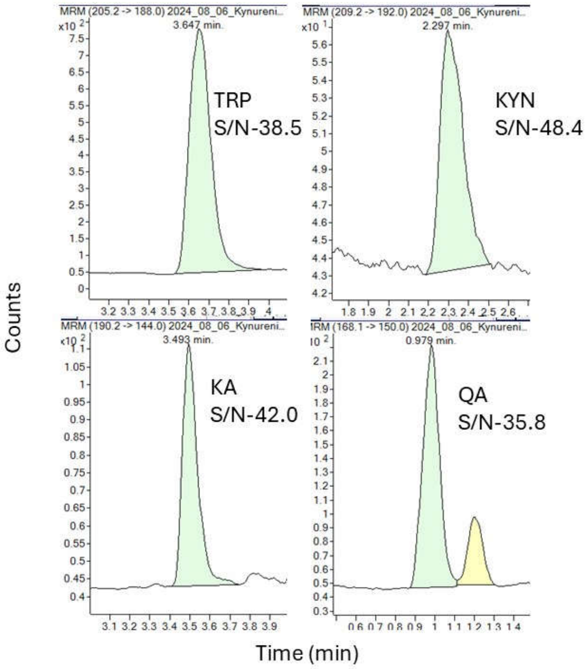

Supplementary Figure 1. Chromatograms of the peak at the limit of detection for each compound (TRP, KYN, KA and QA) in the method.

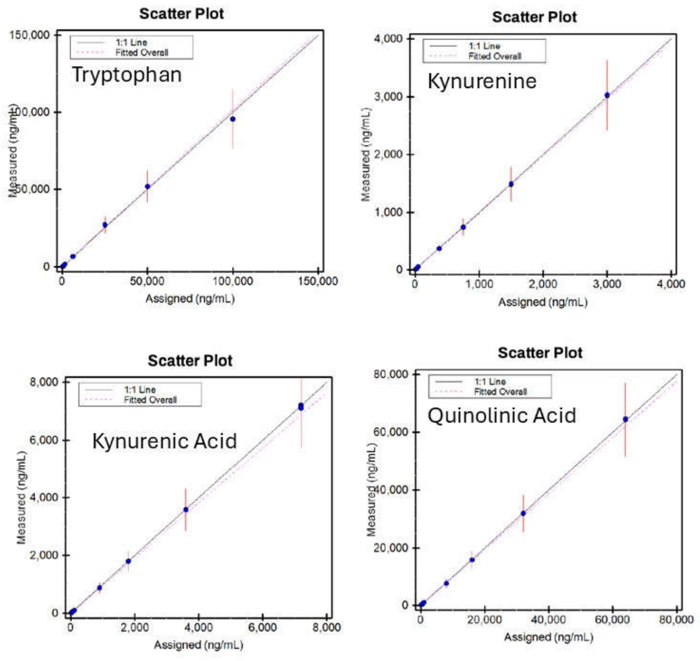

Supplementary Figure 2. Linearity plots from EP Evaluator for each compound (TRP, KYN, KA and QA) in the method.

S. Table 1. Instrument Gradient.

Gradient time (min)	Eluent A (%)	Eluent B (%)
0	95	5
5	40	60
5.01	10	90
6	10	90
6.01	95	5
7	95	5

S. Table 2 List of prescription and illicit compounds evaluated for interference.

Drug Compound	QC Concentration	Unit	Cutoff (ng/mL)	Drug Compound	QC Concentration	Unit	Cutoff (ng/mL)
4-Hydroxyxylazine	1.0	mg/mL	5	Methamphetamine	100	µg/mL	50
6-Acetylmorphine	1.0	mg/mL	10	Methylphenidate	100	µg/mL	50
6-Beta-Naltrexol	100	µg/mL	5	Mirtazapine	100	µg/mL	25
7-Aminoclonazepam	100	µg/mL	25	Mitragynine	100	µg/mL	50
7-Hydroxymitragynine	100	µg/mL	50	Morphine	100	µg/mL	100
9-Hydroxyrisperidone	100	µg/mL	25	Naloxone	100	µg/mL	5
Alpha-Hydroxyalprazolam	100	µg/mL	25	Naltrexone	100	µg/mL	5
Alpha-Hydroxymidazolam	100	µg/mL	25	N-Desmethylcitalopram	100	µg/mL	25
Alpha-Hydroxytriazolam	100	µg/mL	25	N-Desmethylclozapine	100	µg/mL	25
Amitriptyline	100	µg/mL	50	N-Desolanzapine	100	µg/mL	25
Amphetamine	100	µg/mL	50	NN-Dimethylpentaylone	100	µg/mL	5
Aripiprazole	100	µg/mL	25	Norbuprenorphine	100	µg/mL	5
Benzoylecgonine (cocaine metabolite)	100	µg/mL	50	Nordiazepam	100	µg/mL	50
Bromazolam	100	µg/mL	25	Norfentanyl	1.0	mg/mL	3
Buprenorphine	1.0	mg/mL	2	Norfluoxetine	100	µg/mL	100
Carisoprodol	100	µg/mL	50	Norhydrocodone	100	µg/mL	50
Citalopram	100	µg/mL	25	Normeperidine	100	µg/mL	25
Clozapine	100	µg/mL	25	Noroxycodone	100	µg/mL	50
Codeine	100	µg/mL	50	Norquetiapine	100	µg/mL	25

Cotinine	100	µg/mL	25	Nortriptyline	100	µg/mL	25
Cyclobenzaprine	100	µg/mL	100	O-Desmethyl-cis-tramadol	100	µg/mL	50
Dehydroaripiprazole	100	µg/mL	25	O-Desmethylvenlafaxine	100	µg/mL	25
Desipramine	100	µg/mL	50	Olanzapine	100	µg/mL	25
Detomidine	100	µg/mL	50	Oxazepam	100	µg/mL	50
Dextromethorphan	1.0	mg/mL	5	Oxycodone	100	µg/mL	50
Doxepin	100	µg/mL	25	Oxymorphone	100	µg/mL	50
Duloxetine	100	µg/mL	50	Paroxetine	100	µg/mL	25
EDDP Perchlorate	100	µg/mL	50	Pentazocine	100	µg/mL	25
Fentanyl	1.0	mg/mL	2	Pentylone	100	µg/mL	5
Fluoxetine	100	µg/mL	25	Phencyclidine (PCP)	100	µg/mL	10
Gabapentin	10.0	mg/mL	1000	Phentermine	100	µg/mL	50
Haloperidol	100	µg/mL	25	Pregabalin	10.0	mg/mL	1000
Hydrocodone	100	µg/mL	50	Propoxyphene	100	µg/mL	50
Hydromorphone	100	µg/mL	50	Quetiapine	100	µg/mL	25
Hydroxybupropion	100	µg/mL	25	Risperidone	100	µg/mL	25
Imipramine	100	µg/mL	25	Sertraline	100	µg/mL	50
JWH018 5-Hydroxypentyl	100	µg/mL	10	Tapentadol	100	µg/mL	50
JWH250 4-Hydroxypentyl	100	µg/mL	10	Temazepam	100	µg/mL	50
Ketamine	100	µg/mL	50	THC-Delta-9-COOH	100	µg/mL	50
Lamotrigine	100	µg/mL	25	Thebaine	100	µg/mL	2
Levetiracetam	1.0	mg/mL	500	Tianeptine	100	µg/mL	50
Lorazepam	100	µg/mL	100	Tramadol	100	µg/mL	50
Lurasidone	100	µg/mL	25	Trazodone	100	µg/mL	25
MDMA	100	µg/mL	50	Venlafaxine	100	µg/mL	25
MDPV	100	µg/mL	50	Xylazine	100	µg/mL	5
Medetomidine/Dexmedetomidine	100	µg/mL	50	Zaleplon	100	µg/mL	10
Meperidine	100	µg/mL	50	Ziprasidone	100	µg/mL	25
Meprobamate	100	µg/mL	50	Zolpidem	100	µg/mL	10
Methadone	100	µg/mL	50				

S Table 3. Results of the carry-over study.

	**TRP**	**KYN**	**KA**	**QA**
High Cal	93528	3011	7407	65478

Injection 1	0.423	0.025	0.112	2.15
Injection 2	11.6	0.37	0.5	14.9
Injection 3	7.8	0.14	0.16	17.3

S. Table 4 Results of the interference study to determine if blood or protein interfere with results of the assay.

	Expected Result	Sample + Blood	%Bias	Water + Blood
	ng/mL			ng/mL
Tryptophan	67,164	67512	1%	1597
Kynurenic Acid	6,117	5734	-6%	54
Kynurenine	2,303	2269	-1%	247
Quinolinic Acid	25,323	24994	-1%	58

	Expected Result	Sample + BSA		Water + BSA
	ng/mL			ng/mL
Tryptophan	67,164	66419	-1%	Not Detected
Kynurenic Acid	6,117	6455	6%	Not Detected
Kynurenine	2,303	2330	1%	Not Detected
Quinolinic Acid	25,323	27509	9%	Not Detected

Abbreviations: BSA; Bovin Serum Albumin
